# Dentists’ clinical decision-making about teeth with apical periodontitis using a variable-controlled survey model in South Korea

**DOI:** 10.1186/s12903-020-1014-z

**Published:** 2020-01-29

**Authors:** Junghoon Lee, Sumi Kang, Hoi-In Jung, Sunil Kim, Bekir Karabucak, Euiseong Kim

**Affiliations:** 1 Microscope Center, Department of Conservative Dentistry and Oral Science Research Center, Yonsei University College of Dentistry, 50-1 Yonsei-ro, Seodaemun-gu, Seoul, 03722 Republic of Korea; 2 Department of Preventive Dentistry and Public Oral Health, College of Dentistry, Yonsei University, 50-1 Yonsei-ro, Seodaemun-gu, Seoul, 03722 Republic of Korea; 3 Department of Endodontics, School of Dental Medicine, University of Pennsylvania, 240 S 40th St, Philadelphia, PA 19104 USA

**Keywords:** Apical periodontitis, Decision-making, Dentists, Endodontists, Root canal treatment, Survey

## Abstract

**Background:**

This study, by using a variable-controlled survey model, sought to compare clinical decisions made by dentists with different clinical backgrounds in South Korea regarding teeth with apical periodontitis and to identify factors that influenced decision-making.

**Methods:**

A questionnaire with 36 questions about identical patient information, clinical signs, and symptoms was filled out by participants. Each question referred to a radiograph that had been manipulated using computer software in order to control tooth-related factors. Participants were instructed to record their demographic information and choose the ideal treatment option related to each radiograph. Simple and multivariable logistic regression analyses (*p* < .05) were used to investigate factors related to the decision to extract the tooth. We divided factors into dentist-related factors (gender, years of experience, and professional registration) and tooth-related factors (tooth position, coronal status, root canal filling status, and size of the periapical radiolucency). Dentists were categorized into three groups, based on professional registration: general dental practitioners (GDPs), endodontists, and other specialists. Simple logistic regression analysis (*p* < .05) was used to evaluate the tooth-related factors influencing extraction, depending on the dentists’ specialty.

**Results:**

Participants mostly preferred saving the teeth over extraction. This preference was highest among the endodontists, followed by other specialists and GDPs. Extractions were significantly preferred for molars, teeth with previous root canal fillings, and those with apical lesions greater than 5 mm.

**Conclusions:**

This study suggests that dentists’ decision-making regarding teeth with apical periodontitis was associated with their work experience and specialty and influenced by tooth position, root canal filling status, and size of the apical lesion.

**Clinical relevance:**

This survey revealed that clinical decision-making related to teeth with apical periodontitis was affected by dentists’ specialty and work experience and by tooth-related factors, such as tooth position, root canal filling status, and size of the apical lesion.

## Background

Saving teeth is a primary objective in dentistry. Apical periodontitis, an inflammatory lesion around the root apex, is one of the main reasons for tooth extraction [1]. It has a prevalence of one in every three people [2], up to 62% being over 60 years of age [3]. Apical periodontitis is usually managed by root canal treatment. However, if the tooth is difficult to retain because of clinical or other reasons, the clinician may consider extraction. Losing dentition is not only considered a marker of functional aging [4, 5], but also has negative psychological implications for patients [6]. Presence of a few remaining teeth has been associated with a higher prevalence and incidence of dementia [7]. Therefore, patients may prefer preserving teeth affected by apical periodontitis, and this should be dentists’ priority [8].

Several studies show different perspectives among dentists on the treatment planning for apical periodontitis and on factors affecting the decision to extract them [9–12]. Dentists disagree not only about radiographic analyses, but also about treatment decisions in various clinical contexts [12]. In addition, the clinical decision-making may vary depending on the dentist’s clinical background, such as their specialty or current working environment, as well as previous experience [12, 13]. Therefore, the educational or clinical backgrounds that affect dentists’ decision to prefer extraction and the dental factors that are considered while making this decision must be identified. By recognizing the gap in the skills among clinicians, an academic society can provide reasonable prognostic guidelines as well as effective training of skills and knowledge required to conserve teeth.

Previous attempts have compared clinical decisions among various dentist groups using survey models [10–12]. Most of the survey models included examinations of periapical radiographs of patients under a given clinical scenario, after which the clinicians were asked to decide on the most suitable treatment plan. Bigras et al. [10] mailed 5 different clinical scenarios to various dentist groups and discovered that clinical background may affect decision-making among dentists. However, the results did not reveal what dental factors led to the decision to extract, as the model used in the studies did not control for factors of interest, such as periodontal condition, periapical status, quality of previous restoration, or root canal filling status.

Thus, we created a variable-controlled survey model that used a series of radiographs manipulated using a computer graphic program to control some tooth-related factors. The purpose of this study was to compare clinical decisions about apical periodontitis among dentists with different clinical backgrounds in South Korea and to identify factors that influenced different decisions by using a variable-controlled survey model.

## Methods

Eight hundred printed copies of the questionnaire, with a brief cover letter describing the study, were distributed to dentists attending several annual meetings, conferences, and seminars, between September 2017 and March 2018. Verbal informed consents were obtained from all participants because this study was voluntary and the responses were anonymous. In the survey form, the participants were asked to record their demographic information, such as gender, age, year of commencing work as a dentist, and specialty. The survey consisted of 36 questions under the same case scenario, with different variables shown in controlled radiographs.

### Case scenario

For all 36 questions, the identical basic information about the patient was given, as below:

A 43-year-old man presents with the chief complaint, “I have pain while chewing.” The patient did not have any specific medical history. A periapical radiograph was taken of the tooth identified as the origin of pain. The patient felt pain on percussion, and showed no sensitivity to a cold test or electrical pulpal testing. Previous dental restoration was performed more than 5 years earlier at a local clinic. There was no discomfort in the other teeth. Clinically, the tooth showed a normal probing depth and the coronal part was restorable.

### Manipulation of variable-controlled radiographs

**Fig. 1:**
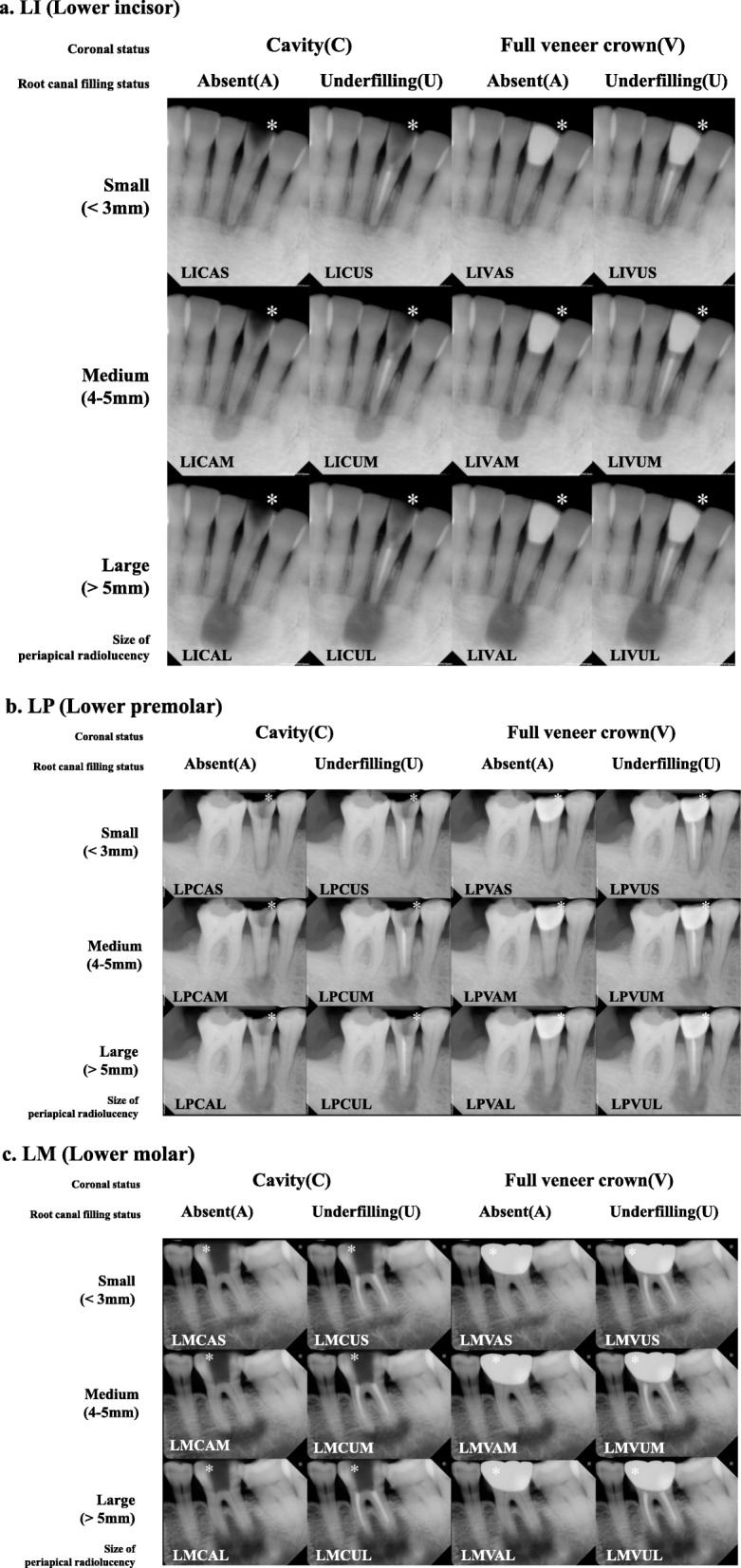
The 36 manipulated radiographs used in this survey. Each radiograph includes the abbreviations described in Table 1

Three base radiographs of a lower incisor, a premolar, and a molar were obtained from the Picture Archiving and Communication System (PACS) of Yonsei University Dental Hospital. The base radiographs showed normal dentition without any clinical defects such as caries, root fractures, root resorption, or radicular lesions. The three original images were manipulated using a computer graphic software package (Adobe Photoshop CS4, Adobe Systems, San Jose, CA, United States) to change tooth-related variables: 2 different coronal states (cavity and full veneered crown); 2 different states of root canal filling (absent and underfilling); 3 different sizes of periapical lesions (small: < 3 mm, medium: 4–5 mm, and large: > 5 mm diameter). Therefore, 36 variable-controlled radiographs were created, as shown in Fig. 1. The tooth-related variables are summarized in Table 1.

**Table 1:** Abbreviations of tooth-related variables (tooth-related factors) for coding the 36 cases in this survey

Variables	Abbreviations		
Tooth position	LI: Incisor	LP: Premolar	LM: Molar
Coronal status	C: Cavity	V: Full veneer crown	
Root canal filling status	A: Absent	U: Underfilling	
Size of periapical radiolucency (diameter)	S: Small (< 3 mm)	M: Medium (4-5 mm)	L: Large (> 5 mm)

The participants were asked to choose their preferred clinical decision from 5 given options for each case scenario:

1. Nonsurgical root canal treatment (or retreatment)
2. Apical surgery
3. Intentional replantation
4. Extraction
5. Refer to specialists

### Statistical analysis

The respondents’ answers were collected and grouped as either “Save or Refer” (answer 1, 2, 3, and 5) or “Extraction” (answer 4). We divided factors into dentist-related and tooth-related factors for clarity. Dentist-related factors included gender, years of experience, and professional registration, while tooth-related factors were tooth position, coronal status, root canal filling status, and size of the periapical radiolucency. Simple and multivariable logistic regression analyses were used to investigate the factors predisposing to opting for extraction.

Dentists were categorized into three groups, based on professional registration: general dental practitioners (GDPs), endodontists, and other specialists (specialists other than endodontists). Simple logistic regression analysis evaluated tooth-related factors influencing extraction, depending on the dentists’ specialty.

For analysis of the extraction rate of each group of dentists in terms of the size of periapical radiolucency, further simple logistic regression analysis was conducted, depending on tooth position, which was divided into incisor/premolar and molar. All statistical analyses were performed using SPSS version 23.0 (IBM; Chicago, IL, USA).

## Results

**Fig. 2:**
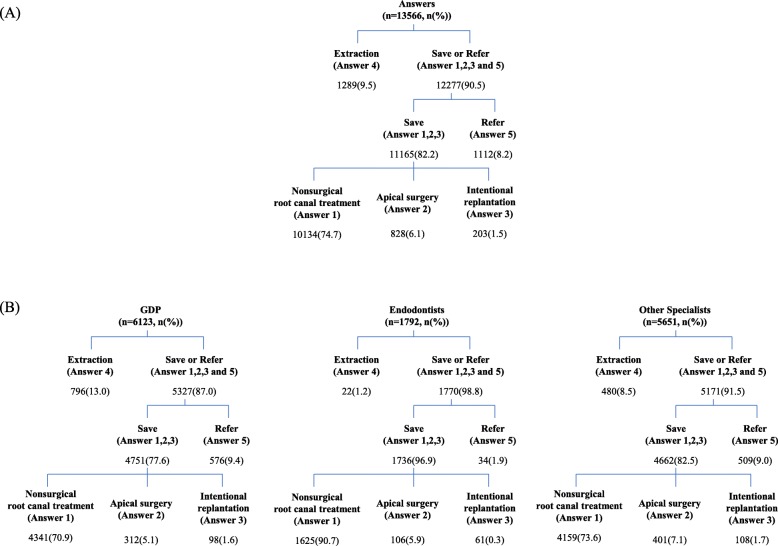
Percentage of participants’ answers to the questions in the survey. **a** A flowchart of categorization of the answers (n(%)). **b** Answers from each group of dentists depicted as a flowchart (n(%))

The survey was completed by 380 dentists (response rate: 47.5%) including 13,566 answers; 114 missing or inappropriate responses were excluded from the data. The demographic characteristics of the participants are summarized in Table 2. Responses from most dentists were grouped into “Save or Refer” (90.5%) compared to “Extraction” (9.5%) for all scenarios. The extraction ratio was the highest among GDPs (13.0%), followed by other specialists (8.5%), and it was the lowest among endodontists (1.2%). The percentages of each answer are shown in Fig. 2.

**Table 2:** Description of the dentists who participated in the survey

	Number of participants (percentage)
Gender	
Female	98 (25.8)
Male	282 (74.2)
Years of experience	
< 5	109 (28.7)
6–15	93 (24.5)
16–25	65 (17.1)
> 25	113 (29.7)
Professional registration	
General dental practitioners	172 (45.3)
Endodontists	50 (13.2)
Other specialists	158 (41.6)
Oral Surgeons	38 (10)
Prosthodontists	36 (9.5)
Periodontists	31 (8.2)
Others^a^	53 (13.9)
Total	380 (100)

^a^Others include pedodontists, orthodontitsts, oral pathologists and oral medicine specialists

To identify factors for extraction, we performed simple and multivariable logistic regression analyses for dentist-related and tooth-related factors (Table 3). Among dentist-related factors, gender showed no significant impact. Dentists who had worked for 6–15 years or more than 25 years were more likely to extract teeth than those who had worked for less than 5 years. In terms of professional registration, oral maxillofacial surgeons showed no significantly greater preference for extraction than GDPs, whereas prosthodontists, periodontists, and other specialists showed significant preferences for saving teeth. Among tooth-related factors, tooth position, root canal filling status, and size of periapical radiolucency affected the clinicians’ treatment decisions. With regard to tooth position, the preference for extraction of molars was significantly higher than that of incisors, whereas the preference for extraction of premolars did not differ significantly compared to incisors. Coronal status did not affect the tendency for extraction. For root canal filling status, previously underfilled teeth showed significantly higher extraction rate than those without history of root canal treatment. With size of periapical radiolucency, extraction rate of teeth with medium and large sized lesion was significantly higher than those with small sized lesion.

**Table 3:** Results from simple and multivariable logistic regression analyses of extraction answers depending on dentist-related and tooth-related factors (*p* < .05)

Variable	n	%	Crude OR^a^ (95% CI)	*P* value	Adjusted OR^b^ (95% CI)	*P* value
Dentist-related factors						
Gender						
Female	347	9.9	Ref.			
Male	955	9.5	1.0 (0.8–1.1)	0.496	1.1 (1.0–1.3)	0.111
Years of experience						
< 5	309	8.0	Ref.			
6–15	287	8.6	1.1 (0.9–1.3)	0.330	1.4 (1.1–1.7)	0.000
16–25	143	6.1	0.8 (0.6–0.9)	0.007	1.1 (0.8–1.3)	0.805
> 25	563	14.0	1.9 (1.6–2.2)	0.000	2.2 (2.0–2.6)	0.000
Professional registration						
General dental practitioners	798	13.0	Ref.			
Endodontists	155	11.4	0.9 (0.7–1.0)	0.110	0.9 (0.7–1.0)	0.141
Oral surgeons	22	1.2	0.1 (0.1–0.1)	0.000	0.1 (0.0–0.1)	0.000
Prosthodontists	137	10.6	0.8 (0.7–1.0)	0.017	0.8 (0.6–0.9)	0.006
Periodontists	81	7.3	0.5 (0.4–0.7)	0.000	0.5 (0.4–0.7)	0.000
Others^c^	109	5.8	0.4 (0.3–0.5)	0.000	0.4 (0.3–0.5)	0.000
Tooth-related factors						
Tooth position						
Incisor	373	8.2	Ref.			
Premolar	352	7.8	0.9 (0.8–1.1)	0.431	0.9 (0.8–1.1)	0.405
Molar	577	12.8	1.6 (1.4–1.9)	0.000	1.7 (1.5–2.0)	0.000
Coronal status						
Cavity	671	9.9	Ref.			
Full veneer crown	631	9.3	0.9 (0.8–1.0)	0.205	0.9 (0.8–1.0)	0.206
Root canal filling status						
Absent	499	7.3	Ref.			
Underfilling	803	11.9	1.7 (1.5–1.9)	0.000	1.8 (1.6–2.0)	0.000
Size of periapical radiolucency						
Small	107	2.4	Ref.			
Medium	290	6.4	2.8 (2.2–3.5)	0.000	2.8 (2.2–3.5)	0.000
Large	905	19.9	10.1 (8.3–12.4)	0.000	10.4 (8.5–12.8)	0.000

*Abbreviations*: *CI* Confidence interval, *OR* Odds ratio, *Ref.* Reference category

^a^Crude OR is the odds ratio resulted from simple logistic regression analysis; ^b^ Adjusted OR is the odds ratio resulted from multivariable logistic regression analysis

^c^Others include pedodontists, orthodontitsts, oral pathologists and oral medicine specialists

**Fig. 3:**
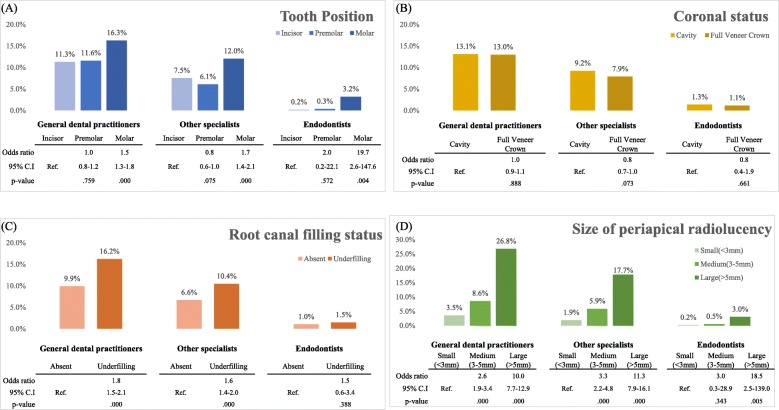
The extraction rate for each group of dentists regarding tooth-related factors **a** tooth position, **b** coronal status, **c** canal filling status, and **d** size of periapical radiolucency. ‘Ref.’ indicates the reference category of each factor (simple logistic regression analysis, *p* < .05))

We also conducted simple logistic regression analysis for extraction rate of each group of dentists (GDPs, endodontists, and other specialists) regarding tooth-related factors (tooth position, coronal status, canal filling status, and size of periapical radiolucency) (Fig. 3). Dentists in all groups were more likely to extract molars than incisors. The larger the size of the periapical radiolucency, the greater was the tendency for extraction. However, the presence of root canal filling material significantly increased the tendency for extraction among GDPs and other specialists, but not among endodontists.

**Fig. 4:**
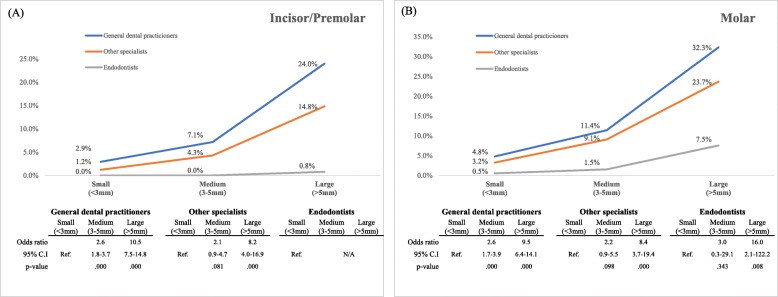
Extraction rate for each group of dentists regarding size of periapical radiolucency. Cases of incisors and premolars (**a**) and those of molars (**b**) are depicted separately for describing different results from the tooth position. ‘Ref.’ and ‘N/A’ indicate the reference category and ‘not applicable’, respectively (simple logistic regression analysis (p < .05))

Considering that tooth position (molar) and a large periapical radiolucency size significantly affected it, the extraction rate for each group of dentists was determined using simple logistic regression analysis in terms of the size of the periapical radiolucency by separating teeth into incisors/premolars and molars (Fig. 4). GDPs were more likely to extract teeth as the size of the periapical lesion increased, regardless of tooth position. Endodontists, however, showed different tendencies. For incisors/premolars, when the lesion was small or medium-sized, none of the endodontists preferred extraction. Even for large lesions, only a few endodontists (0.8%) selected extraction. In cases of molars with small or medium-sized periapical lesions, endodontists rarely elected to extract (0.5% for small sized lesion and 1.5% for medium sized lesions). However, in cases of molars with large periapical lesions, a relatively larger number of endodontists (7.5%) chose extraction as the ideal treatment plan.

## Discussion

Previous studies have compared the tendencies in clinical decision-making among dentists by means of surveys that included periapical radiographs [10–12]. Although Azarpazhooh et al. excluded radiographs from their survey because of considerable inter- and intra-observer variability [9], radiographs are crucial when making clinical decisions. A previous study used line drawings of simulated radiographs of a central incisor, which varied in terms of the quality of the root filling and the presence or absence of a root canal-retained post with crown and periapical conditions [14]. This study was similar to ours in terms of usage of consistent image design, which allows controlled evaluations. However, that study used quality of seal, post, and periapical conditions as tooth-related factors, while we considered tooth position, coronal status, root canal filling status, and size of periapical radiolucency. Our consideration was based on the American Association of Endodontists (AAE) Endodontic Case Difficulty Assessment Form and Guidelines because the conditions listed in this form are potential risk factors that may complicate treatment and adversely affect outcomes [15]. Additionally, the previous study used schematic images of incisors with variations of tooth-related factors, while we modified actual periapical radiographs of incisors, premolars, and molars, thus making our study more clinically relevant. This study was thus unique in terms of inclusion of not only radiographs, but also standard tooth-related control factors.

In this study, we employed a variable-controlled survey model in which periapical radiographs were manipulated to control for some tooth-related factors. The 36 manipulated images were derived from three original images of different tooth positions (incisor, premolar, and molar), using a computer program for the variables mentioned in Table 1. For simplicity and understanding of the variable-controlled radiographs in Fig. 1, we labeled each image using the abbreviations listed in Table 1. For example, LMVUL is a lower molar with a previously underfilled tooth, full veneered crown, and large-sized apical periodontitis. LMVUS, LMVUM, and LMVUL all represent the same lower molar tooth with a previously treated, full veneered crown, but with different sizes of periapical radiolucency. In this manner, we created images in which only one factor varied. Previous surveys on variability in decision-making among dentists compared pairs of inconsistent clinical radiographs [10–12]. Consequently, these studies lacked evidence for deriving dental factors that are associated with case difficulty. With our study design, we were able to perform multivariable logistic regression analysis and more accurately identify the influencing tooth-related factors.

The response rate of this study was 47.5%. Most of the non-responders did not participate for no specific reason or for non-relatability to their specialty (oral medicine, orthodontics, and so forth). Participation was partly affected due to the absence of a reward. Unfortunately, we could not conduct drop-out analysis because of limited information on the years of clinical experience and specialty of the nonresponders; only 5% of them (21 of 420 nonresponders) provided this information. However, in terms of the included study population, we attempted to conduct this survey in an uncontrolled manner and included dentists at several meetings for continuing dental education. Thus, we assume that the sample, i.e., those who attended these meetings, were representative of actively working dentists, which implies that they are the key clinical decision makers. In this context, the study population was acceptable for the purpose of this survey.

In all 36 cases presented in the questionnaire, the teeth were classified as having symptomatic apical periodontitis with pulp necrosis or as a previously treated tooth, according to the clinical classification of pulpal and periapical disease by the AAE [16, 17]. This classification is closely related to the clinical treatment plan. Primary endodontic disease should be treated solely through nonsurgical endodontic treatment, and the prognosis is generally favorable [15]. The results of this study indicate that dentists mostly prefer to save teeth affected by apical periodontitis (Fig. 2). However, the percentage choosing extraction varied with case and dentists’ group.

GDPs tended to choose extraction significantly more often than did other specialists, and endodontists had the strongest tendency toward saving teeth (Table 3). This tendency has also been noted in other studies. A study by Pagonis et al. [18] comparing retreatment decisions between GDPs and endodontic postgraduates concluded that GDPs were more likely to initiate extensive treatment early. Other studies in Greece [11] and the United Kingdom [12] noted that general dentists and undergraduates establish inconsistent agreement or radical judgment as compared with endodontists and postgraduate students. In Azarpazhooh et al.’s well-controlled study, GDPs in Ontario chose extraction over root canal treatment four times more often than did endodontists (15.7% versus 4.1%, pooled data) [9]. Their study differed from ours in that other specialists in Ontario tended to extract (32.0%, pooled data) markedly more often than did the GDPs; in our study, other specialists chose extraction less often than GDPs. This difference could be explained by variations in working environments. As prosthodontists and periodontists usually work as advanced GDPs in Korea, they also perform a marked number of endodontic treatments, whereas specialists in Ontario rarely offer endodontic treatment as part of their dental service.

Years of experience was a factor affecting dentists’ decision-making. Dentists with more than 25 years of clinical experience were 2.3 times more likely to extract than those with less than 5 years of experience (Table 3). This may imply that experienced dentists rely more on their clinical experience when making such decisions. There was no significant difference between male and female dentists (Table 3). Another study reported that male dentists performed a higher percentage of extractions, but this factor was not as significant as their specialty [9]. Therefore, whether the gender of the dentist influences decision-making is not clear.

Decisions related to molars differed significantly from those related to incisors and premolars. This result is consistent with the AAE assessment form explaining tooth position factors, in which root canal treatment of molars is considered moderately difficult and is recommended to be performed by competent, experienced practitioners to obtain a good outcome, whereas the same treatment of an incisor or premolar is considered to pose minimal difficulty. Several studies have also shown lower success rates for root canal treatment of molars than for incisors and premolars [10, 19, 20] and high referral rates to endodontists [21]. Limited accessibility or multiple visits might be obstacles to root canal treatment of molars. In addition, teeth with marginal periodontitis can pose moderate to high difficulty for achieving a favorable treatment outcome and thus could be one of the reasons for the higher extraction rate of molars [15].

GDPs and other specialists chose extraction in a previously root canal-treated tooth (Fig. 3). In contrast, previous endodontic treatment did not affect the endodontists’ decisions. A history of surgical or nonsurgical endodontic treatment is considered a high-difficulty factor [15] in the AAE assessment form, which then recommends considering referral to endodontists. Several studies have found that endodontists perform more successful endodontic retreatments than GDPs [19, 21]. Thus, GDPs and other specialists should consider referral to endodontists for such challenging cases, rather than opting for extraction.

As the size of periapical radiolucency increased, the extraction ratio also increased among GDPs (Fig. 4). This suggests that they believe that a larger lesion affects prognosis detrimentally. However, according to the AAE guidelines, the presence of periapical radiolucency is not an absolute indicator of a poor long-term prognosis, and the size of a lesion is not a factor in case difficulty. A study on endodontic prognosis by Ng et al. found that larger lesions tended to have a poor prognosis, but no statistical significance [10, 22]. In contrast, here, endodontists were confident of preserving the tooth, especially in small and medium lesion size in the incisor or premolar. Although a large apical radiolucency could lower the success rate of nonsurgical endodontic treatment, the endodontists considered apical surgery or intentional replantation. Moreover, as the paradigm has shifted from traditional endodontic surgery to endodontic microsurgery (EMS) using a dental microscope and bioceramics [23], surgical endodontic retreatment has become a reliable treatment option when nonsurgical endodontic treatment fails. However, in cases involving previously treated molars, the tendency towards extraction increased, even among endodontists, because of the difficulty quotient for endodontic retreatment, even with loupes or dental microscopes.

The results of this study mirror the clinical situation in current dentistry, where, even though endodontists are capable of saving a tooth with apical periodontitis, some GDPs are more likely to extract the tooth rather than save it or refer the patient to specialists. Interestingly, dentists who made such decisions ranked the decision-making process as easy [10]. Moreover, the majority of dentists thought that their colleagues would make a similar decision [24]. A consensus has not been reached despite academic associations’ efforts to increase clinical agreement regarding the treatment of apical periodontitis, and GDPs continue to rely on their clinical experience and follow a fast and extensive treatment plan [18]. Patients universally prefer to save their painful teeth over extraction [8]. A clinician’s duty is to offer the best care to patients and cater to their preferences within the scope of treatment; therefore, continuous and proper education should be offered to GDPs to provide better dental care.

## Conclusions

This survey investigated factors that affect dentists’ decision-making regarding teeth with apical periodontitis. Among dentist-related factors, work experience, and dentists’ specialty were associated with decision-making. GDPs decided on extraction more often than did specialists. Among tooth-related factors, tooth position, root canal filling status, and size of apical lesion influenced the clinical decision-making of GDPs and other specialists more than that of endodontists.

## Data Availability

The datasets used and/or analysed during the current study are available from the corresponding author on reasonable request.

## Abbreviations

AAE: American Association of Endodontists
GDPs: General dental practitioners
PACS: Picture Archiving and Communication System
